# The complete mitochondrial genome of the early flowering plant *Nymphaea colorata* is highly repetitive with low recombination

**DOI:** 10.1186/s12864-018-4991-4

**Published:** 2018-08-14

**Authors:** Shanshan Dong, Chaoxian Zhao, Fei Chen, Yanhui Liu, Shouzhou Zhang, Hong Wu, Liangsheng Zhang, Yang Liu

**Affiliations:** 1 Fairylake Botanical Garden, Shenzhen & Chinese Academy of Sciences, Shenzhen, China; 2 College of Life Sciences, South China Agricultural University, Guangzhou, China; 3 Department of Biology, School of Life Sciences, East China Normal University, Shanghai, China; 4 State Key Laboratory of Ecological Pest Control for Fujian and Taiwan Crops, Fujian Agriculture and Forestry University, Fuzhou, China; 5 Ministry of Education Key Laboratory of Genetics, Breeding and Multiple Utilization of Corps, Fujian Agriculture and Forestry University, Fuzhou, China; 6 Fujian Provincial Key Laboratory of Haixia Applied Plant Systems Biology, Fujian Agriculture and Forestry University, Fuzhou, China; 7 BGI-Shenzhen, Shenzhen, 518083 China

**Keywords:** Mitochondrial genome, *Nymphaea*, Repeats, Recombination, PacBio sequencing

## Abstract

**Background:**

Mitochondrial genomes of flowering plants (angiosperms) are highly dynamic in genome structure. The mitogenome of the earliest angiosperm *Amborella* is remarkable in carrying rampant foreign DNAs, in contrast to *Liriodendron*, the other only known early angiosperm mitogenome that is described as ‘fossilized’. The distinctive features observed in the two early flowering plant mitogenomes add to the current confusions of what early flowering plants look like. Expanded sampling would provide more details in understanding the mitogenomic evolution of early angiosperms. Here we report the complete mitochondrial genome of water lily *Nymphaea colorata* from Nymphaeales, one of the three orders of the earliest angiosperms.

**Results:**

Assembly of data from Pac-Bio long-read sequencing yielded a circular mitochondria chromosome of 617,195 bp with an average depth of 601×. The genome encoded 41 protein coding genes, 20 tRNA and three rRNA genes with 25 group II introns disrupting 10 protein coding genes. Nearly half of the genome is composed of repeated sequences, which contributed substantially to the intron size expansion, making the gross intron length of the *Nymphaea* mitochondrial genome one of the longest among angiosperms, including an 11.4-Kb intron in *cox2*, which is the longest organellar intron reported to date in plants. Nevertheless, repeat mediated homologous recombination is unexpectedly low in *Nymphaea* evidenced by 74 recombined reads detected from ten recombinationally active repeat pairs among 886,982 repeat pairs examined. Extensive gene order changes were detected in the three early angiosperm mitogenomes, i.e. 38 or 44 events of inversions and translocations are needed to reconcile the mitogenome of *Nymphaea* with *Amborella* or *Liriodendron*, respectively. In contrast to *Amborella* with six genome equivalents of foreign mitochondrial DNA, not a single horizontal gene transfer event was observed in the *Nymphaea* mitogenome.

**Conclusions:**

The *Nymphaea* mitogenome resembles the other available early angiosperm mitogenomes by a similarly rich 64-coding gene set, and many conserved gene clusters, whereas stands out by its highly repetitive nature and resultant remarkable intron expansions. The low recombination level in *Nymphaea* provides evidence for the predominant master conformation in vivo with a highly substoichiometric set of rearranged molecules.

**Electronic supplementary material:**

The online version of this article (10.1186/s12864-018-4991-4) contains supplementary material, which is available to authorized users.

## Background

The advent of high-throughput sequencing technologies has greatly promoted the research for plant mitochondrial (mt) genomes. Most (~ 80%, 176 out of 214) of the plant mitogenomes deposited in the GenBank database (https://www.ncbi.nlm.nih.gov/genome/organelle/) were generated in the past several years (since 2011). Research of plant mitogenomes have also been expanded to cover phylogenetically more diverse organisms from focusing on crops [1]. To date (as of July 2018), 53 bryophyte and 108 vascular plant complete mitogenomes have been reported (https://www.ncbi.nlm.nih.gov/genome/organelle/). Among them, bryophyte mitogenomes show astoundingly conserved structures and stable genome content in its major lineages [2–4], whereas vascular plant mitogenomes vary significantly in both genome structure and content [1, 5], nucleotide substitution rates [6–8], and repeat recombination level [1, 9]. In particular, angiosperm mitogenomes exhibit highly dynamic characters: ranging from 66 Kb [10] to 11.3 Mb [7] with 19 to 64 [10] known genes (not including duplicate genes and ORFs), 5 [10] to 25 [1] introns, and highly variable intergenic regions [11]. The ca. 200-fold range of mitogenome size divergence is primarily due to the variation in non-coding regions, including repeated sequences [12], introns [13], intracellular transferred sequences from plastid [14] and nucleus [13], and horizontal gene transfers from foreign donors [15, 16].

Vascular plant mitogenomes sequenced to date generally contain a large fraction of repeated sequences of unknown origin [1], with some genomes including numerous short dispersed repeats < 100 bp (e.g., *Cucurbita pepo* [14], *Cycas taitungensis*, [17]), some containing considerable amount of large repeats > 1000 bp (e.g., *Oryza sativa*, [18], *Zea mays subsp. parviglumis* [19]), and some both (e.g., *Silene conica*, [7], *Psilotum nudum* [9]). The prevalence and activity of the repeated sequences play a pivotal role in shaping the plant mitogenome structure [20, 21] through participation in pervasive genome rearrangements [22], recombination dependent replication initiations [23], genome sequence duplications, inversions, insertions and deletions [24]. Up to now, mitochondrial homologous recombinations involving repeated sequences have been investigated in about fourteen vascular plant species with high-depth sequencing data [1, 7, 9, 13, 25–32]. Particularly, studies employing quantitative measuring methods unequivocally uncovered positive correlations between repeat length and recombination rate [10]. Although most of these studies detected minor to moderate recombination activities among small (< 100 bp) and medium sized repeats (100~ 1000 bp) [7], large repeat (> 1000 bp) mediated recombinational equilibrium was also frequently observed in a number of species, including the angiosperms *Mimulus guttatus* [33], *Silene latifolia* [32], *Silene vulgaris* [7], *Cucumis sativus* [13], and the gymnosperm *Ginkgo biloba* [31]. Recently, the third-generation long-read DNA sequencing technologies have yielded high quality assemblies for plant mitogenomes, which enabled more accurate and sensitive detection for homologous recombinations [30], apparently devoid of false positives introduced by PCR artifacts [20] or insufficient read length in the Next Generation Sequencing (NGS) approaches [10].

Study of early angiosperm mitogenomes would improve the entire view on the evolutionary pattern of plant mitogenomes. Two available mitogenomes of the earliest angiosperms *Amborella* [16] and *Liriodendron* [6] show a series of distinctive features. The 3.9-Mb mitochondrial genome of *Amborella* with a 63 coding gene set, houses massive horizontal gene transfers (HGTs) from a variety of organisms [16], which is unparalleled and extremely unusual, considering the sporadic occurrences of HGTs detected in some vascular plant mitogenomes, such as *Gnetum* [34], *Malpighiales* [35], *Plantago* [36], *Viscum* [37] and *Lophophytum* [38]. The 553-Kb mitogenome of *Liriodendron* with a similar 64 coding gene set, is otherwise described as “fossilized” due to its extremely low synonymous substitution rate, retention of genes that are missing in the other lineages and many ancestral gene clusters [6]. An expanded sampling of the early angiosperm mitogenomes is needed to elucidate the distribution pattern of these features in early angiosperm mitogenomes. *Nymphaea* (Nymphaeaceae, Nymphaeales), commonly known as water lilies, hold a critical evolutionary status for understanding the origin and early evolution of flowering plants [39]. This pantropical genus belongs to the most species-rich, early diverging flowering plant order Nymphaeales [40], which are deemed as “the first globally diverse clade” [41] within extant angiosperms, compared with the other two early angiosperm lineages, Amborellales and Austrobaileyales, both with limited distribution ranges [42]. In phylogenetic studies, Nymphaeales were resolved as a member of the “ANITA” (*Amborella*, Nymphaeales, and Illiciales-Trimeniales-Aristolochiales) clades [43], either forming a cluster with *Amborella* at the base of angiosperms [44–46], or diverging after *Amborella* as the second paraphyletic lineage of angiosperms [47].

In this study, we presented the complete mitogenome of *Nymphaea colorata* Peter, a tropical water lily from East Africa [39] to investigate the mitogenomic evolution of early flowering plants. The 617,195-bp mitogenome of *Nymphaea* encoded a similar 64 coding gene set with 25 group II introns disrupting 10 protein-coding genes, comparable to the other two early angiosperms such as *Amborella* and *Liriodendron*. Our study pinpointed the highly repetitive nature of *Nymphaea*, the resultant remarkable intron expansions in *Nymphaea* mitogenome, but unexpectedly low homologous recombination.

## Results and Discussions

### General features of *Nymphaea* mitogenome

**Fig. 1:**
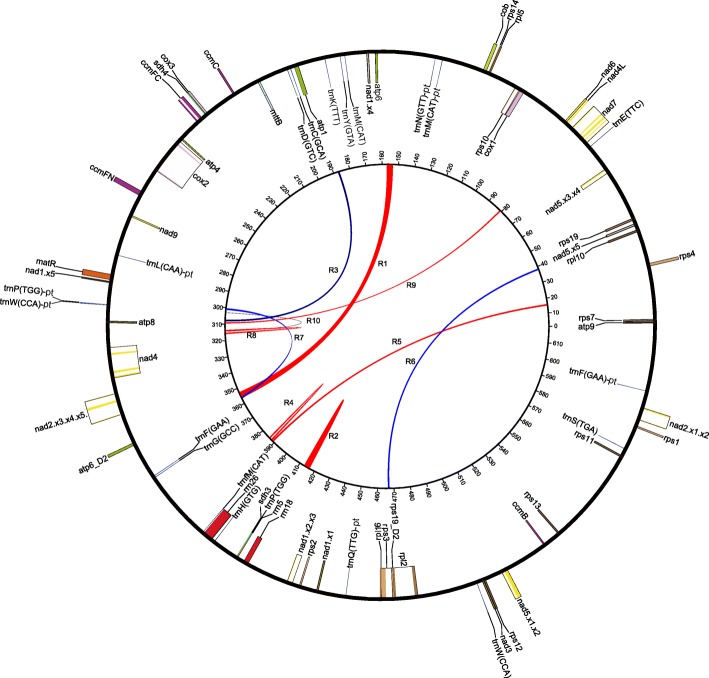
Mitochondrial genome map of *Nymphaea colorata*. The total length of the *Nymphaea* mitogenome is 617,195 bp. Genes (exons are shown as closed boxes) shown on the outside of the circle are transcribed clockwise, whereas those on the inside are transcribed counter-clockwise. Genes from the same protein complex are colored the same, introns are indicated in white boxes, and tRNAs of plastid origin are noted with a ‘-pt’ suffix. The inner circle shows the locations of direct (blue) and inverted (red) repeats (R1 to R10) with evidence for recombination activity (see Methods and Table 2). Numbers on the inner circle represent genome coordinates (Kb)

The *Nymphaea* mitogenome is assembled into a single circular molecule of 617,195 bp (Fig. 1), a size larger than ca. 80% of the currently sequenced vascular plant mitogenomes (as of July 2018). The relatively large size of *Nymphaea* mtDNA is primarily due to its abundant repetitive sequences, which add up to 301,676 bp and account for nearly half (49%) of the mitogenome, in contrast to most of other vascular plant mitogenomes with repeat ratio generally below 30% (Additional file 1: Table S1). The *Nymphaea* mitogenome encodes 41 protein genes, three rRNA genes (*rrn5*, *rrn18* and *rrn26*), and 20 tRNA genes (13 mitochondrial native and seven plastid derived) (Table 1). Intergenic spacers constitute the largest part (519,361 bp, 84%) of the *Nymphaea* mtDNA, and protein coding sequences comprise only 6% (35,961 bp) of the total length. In general, the gene content of *Nymphaea* is very similar to the other published angiosperm mitogenomes, especially to *Amborella* [48] and *Liriodendron* [6]. *Nymphaea* mt gene set differs from *Amborella* only by its presence of the functional protein-coding gene *rps10* that is pseudogenized in *Amborella*, whereas differs from *Liriodendron* by its presence of plastid derived tRNA gene *trnL*(*CAA*)*-*pt and absence of *trnV*(*TAC*). Repeat-induced duplicated genes are widespread in vascular plants [49], such as *Nelumbo nucifera* possesses six duplicated protein genes [50] and maize (CMS-C) contains 10 duplicated protein genes [51]. In *Nymphaea* mitogenome, *rps19* and *atp6* each presents as two copies. The duplicated *rps19* are identical, while the two copies of *atp6* are different in length, with one copy 114 bp longer at the 3′ terminal. The shorter version of *Nymphaea atp6* is still longer than that of *Amborella* and *Liriodendron* by 36 bp and 75 bp at the 5′ terminal. Blastn and Blastp searches of the 114-bp nucleotide sequence and the translated amino acid sequence against the NCBI database do not return any hits, suggesting a probably chimeric origin of *atp6_D2* (the longer copy), via gene fusion of *atp6* with *Nymphaea* specific intergenic spacer sequence at some evolutionary stage. Considering the majority of the two *atp6* copies located in a pair of identical inverted repeats of 3293 bp at a distance of 196 Kb, the identical 882 bp of the two *atp6* copies may be indicative of the result of repeat recombination in homogenization of the gene copies carried [7]. We further checked all intergenic spacers for possible pseudogene pieces using 68 annotated *Nymphaea* coding regions as queries. Altogether, we identified 52 pseudogene fragments ranging from 28 bp to 182 bp, which matched nine protein coding genes (*nad5.× 2.× 5*, *rpl2.× 1*, *co× 1*, *atp6*, *ccmC*, *nad6*, *atp8*, *rrn18*, *rrn26*) with identities ranging from 85 to 100%. Two largest pseudogene pieces of *atp6* (182 bp) and *rpl2.× 1* (142 bp) formed *Nymphaea* specific chimeric ORFs with parts of the adjacent intergenic spacer sequences, which, in some cases, may cause cytoplasmic male sterility (CMS) [52]. Blastn search of all these pseudogene fragments against the NCBI nucleotide database yielded much lower similarities with any other species than *Nymphaea*, indicating the origin of these gene vestiges from intragenomic recombination events [25] rather than horizontal gene transfers from other plants.

**Table 1:** General features of mitochondrial genomes of *Amborella*, *Liriodendron*, and *Nymphaea*

Genome feature	*Amborella trichopoda*	*Nymphaea colorata*	*Liriodendron tulipifera*
Accession	KF754799–KF754803	KY889142	KC821969
Size (bp)	3,866,039	617,195	553,721
GC%	45.9%	45.1%	47.7%
Genes	63	64	64
tRNAs	20	20	20
rRNAs	3	3	3
Protein coding	40	41	41
Cis-spliced introns	19	19	20
Trans-spliced introns	6	6	5
Gross length of repeats (> 19 bp)	914,403 (23.65%)	301,777 (48.89%)	85,993 (15.53%)
Plastid-derived (bp)	138,313 (3.58%)	13,170 (2.13%)	24,807 (4.48%)
Total gene length (bp)	85,152 (2.20%)	97,834 (15.85%)	82,284 (14.86%)
Protein exons (bp)	33,866 (0.88%)	35,961 (5.83%)	35,324 (6.38%)
Cis-spliced intron length (bp)	38,896 (1.01%)	54,840 (8.89%)	33,631 (6.07%)

*Nymphaea* shares 27% (168,686 bp) of its mtDNA with other sequenced plant mitogenomes with nearly half occurred in the genic region, and the other half (95,941 bp) in the intergenic region, accounting for 15% of the mitogenome. *Nymphaea* shares its intergenic spacer sequences the most with *Amborella* (58,049 bp), and *Liriodendron* (28,715 bp), then *Phoenix* (26,790 bp). As multiple lines of evidence suggested a divergence time of *Nymphaea* from the rest of angiosperms at 180 Mya (www.timetree.org), the seemingly low level of sequence sharing between *Nymphaea* and other angiosperms fits well to the regression line generated by analyzing 14 phylogenetically independent seed plant taxa [31], suggesting the generally high divergence nature of angiosperm mitogenomes. For example, *Citrullus lanatus* [14] shares with *Vitis vinifera* [49] 72,313 bp of its intergenic spacers despite a divergence time of 105–115 Mya; *Carica papaya* [53] shares with *Nicotiana tabacum* [54] 66,327 bp with a divergence time of 110–124 Mya.

**Fig. 2:**
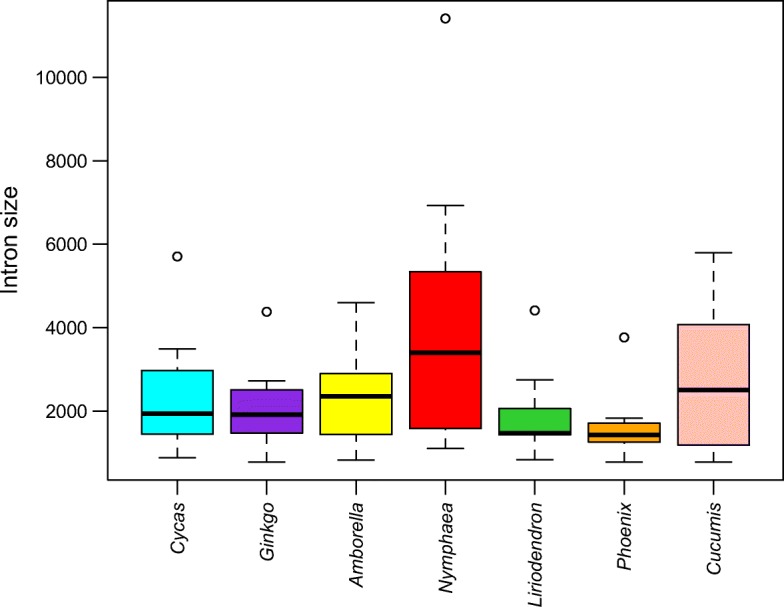
Comparison of the length of 11 introns (see Additional file 4: Table S2) of the *Nymphaea* mitogenome with repeated sequence inserted with that of some selected seed plant mitogenomes

The *Nymphaea* mitogenome contains 25 group II introns, including 19 cis-spliced and six trans-spliced introns (*nad1i394g2*, *nad1i669g2*, *nad1i728g2*, *nad2i542g2*, *nad5i1455g2*, *nad5i1477g2*), which is similar to the intron set of *Amborella* [48] and *Phoenix* [55], but differs from *Liriodendron* by its presence of the trans-splicing *nad1i728g2*, which is a cis-spliced intron in *Liriodendron*. It is noteworthy that *cox2i373g2* of *Nymphaea* reaches a length of 11.4 Kb, making it the longest organellar intron reported in plants to date. We checked the coverage of the genome assembly on this intron region. A continual and even coverage of *cox2i373* and its *cox2* exon regions indicated that the presence of this intron is unlikely yielded from an artifactual assembly result (Additional file 2: Figure S3). We mapped the transcriptomic reads to the mitogenome, but due to the low coverage of the transcriptome data we cannot figure out whether this intron is continually transcribed (Additional file 2: Figure S3). We aligned the *Nymphaea cox2i373g2* with that of *Triticum timopheevii* [56], five out of the six conserved domains of this group II intron were well aligned, except for the domain IV, indicating this domain may expanded in *Nymphaea* (Additional file 3: Figure S4). Although we recognized *Nymphaea cox2i373* as a cis-spliced intron here, we still cannot rule out the possibility that this intron is trans-spliced, but the two parts of the trans-spliced intron happens to locate proximately in the genome and in an orientation consistent with cis-splicing. Besides, intron *rpl2i846g2* and *nad4i976g2* exceed 6 Kb; intron *nad2i1282g2*, *nad2i156g2*, and *nad7i917g2* exceed 3 Kb in length. Overall, the total length of the 19 cis-spliced introns add up to 55 Kb, comprising 9% of the whole mitochondrial genome, which is substantially higher than any other angiosperm mitogenomes sequenced to date in both absolute and percentage terms [13]. The highly repetitive nature of the *Nymphaea* mtDNA accounts for a large portion of its intron size expansion (Fig. 2). About 40% to 80% of the six large introns of the *Nymphaea* mitogenome (> 3 Kb) are made of repetitive elements, a phenomenon similar to what observed in ferns [9] (Additional file 4: Table S2).

### Repeats and homologous recombinations

Blastn searches identified 1,188,860 repeated sequences that are longer than 30 bp and with unique begin-end coordinates in an overlapping fashion, accounting for nearly half (49%, 301,676 bp) of the *Nymphaea* mitogenome (Additional file 1: Table S1). These numerous imperfect and partially overlapping repeated sequences in *Nymphaea* constitute 886,983 repeat pairs, with the length distribution mainly in the range of 100–200 bp and identity distribution mainly between 80 and 95% (Additional file 5: Figure S1). Cd-hit-est as implemented in the cdhit suite [57] recovered 290 families with an identity threshold of 0.8 and a word size of five out of the total repeated sequences using a greedy incremental clustering algorithm method. The representatives of these repeat families were subsequently checked for occurrences using blastn searches against NCBI nucleotide database. Most (252, 87%) of these repeat families are restricted to *Nymphaea* and are unique in plants, only 38 are shared with other plant mitogenomes, such as 22 with *Amborella*, 18 with *Liriodendron*, 11 with *Arabidopsis*, 11 with *Gymnosperms*, four with ferns, eight with bryophytes, and nine with charophycean green algae. The observed low repeated sequence sharing of *Nymphaea* with other plant mitogenomes reflected a commonplace phenomenon of wild divergence of intergenic spacers as has been exemplified by the remarkable intraspecific variation in four mitogenomes of *Silene vulgaris* [25].

**Fig. 3:**
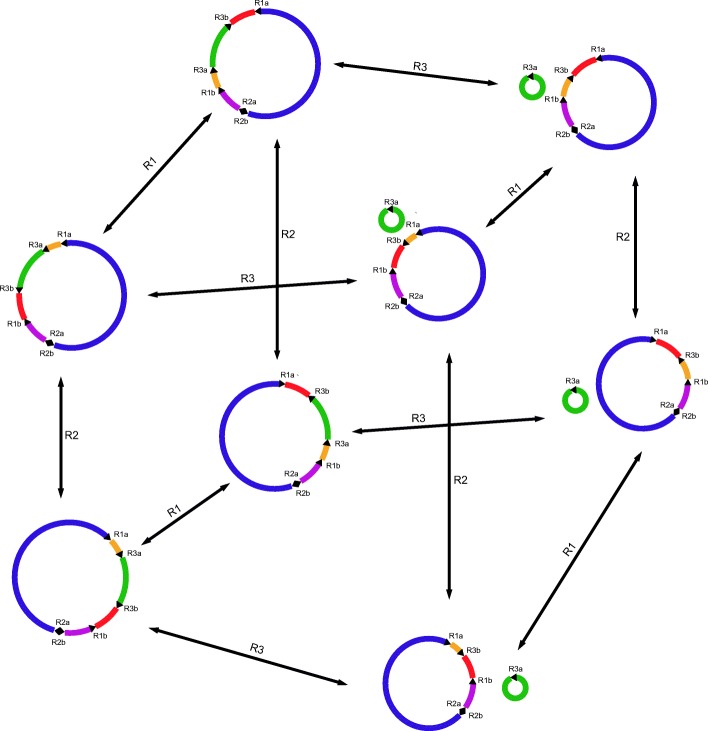
Mitochondrial genome rearrangements and alternative genomic conformations observed from *Nymphaea colorata* based on repeat-mediated intra-molecular recombination products of three repeat pairs (R1, R2 and R3) that induced recombination with the highest frequencies as listed in Table 2

Benefited from the deep sequencing of PacBio long reads (601×, average 7294 bp, Additional file 6: Figure S2), we were able to detect minor recombinations at a frequency as low as 1/1200. A total of 886,983 repeat pairs with length ranging from 30 to 3293 bp and blast identity above 80% were examined for recombination activity (Additional file 5: Figure S1). Unexpectedly, only ten repeat pairs show evidence of recombinations with one to 48 recombined reads detected for each repeat pair (Table 2). Three direct repeats and seven inverted repeats recombined at frequencies ranging from 0.07 to 8.18%, which could possibly give rise to a set of alternative mtDNA configurations and subgenomes via inversions and subdivisions of the master conformations (Fig. 3). According to our observations, a majority of the repeats (R3–R10) recombined at a frequency below 1%; only two repeats yielded more than 10 recombined reads, including the longest inverted repeats of 3293 bp with 48 recombined reads and a self-inverted repeats of 128 bp with 13 recombined reads detected, suggesting alternative conformations (ACs) with a full set of genes rearranged are more abundant than subgenomes with reduced gene set in *Nymphaea* mitochondria, which resembles that observed in fern *Ophioglossum* with predominant ACs harboring inversions induced by the longest 4-Kb inverted repeats recombining at a frequency of 24.5% and a small number of subgenomes generated by recombinationally less active medium-sized repeats recombining at a frequency less than 2.5% [9].

**Table 2:** Recombination frequency of the mitochondrial genome of *Nymphaea colorata* related to ten repeat pairs

Repeat	Length (bp)	Identity (%)	Direction	Position	Reads support master circle conformation	Reads support alternative conformation
R1	3293	100.00	+	153,171–156,463	539 (91.82%)	48 (8.18%)
			–	355,403–352,111		
R2	128	82.81	+	414,017–414,144	1000 (98.72%)	13 (1.28%)
			–	414,144–414,017		
R3	462	100.00	+	187,030–187,491	874 (99.66%)	3 (0.34%)
			+	307,094–307,555		
R4	1538	97.33	+	386,370–387,906	822 (99.76%)	2 (0.24%)
			–	389,671–388,149		
R5	508	81.50	+	15,484–15,960	960 (99.79%)	2 (0.21%)
			–	388,795–388,149		
R6	399	99.75	+	39,755–40,153	880 (99.77%)	2 (0.23%)
			+	466,916–467,314		
R7	945	98.41	+	301,710–302,649	915 (99.89%)	1 (0.11%)
			+	357,890–358,834		
R8	729	99.86	+	313,110–313,838	932 (99.89%)	1 (0.11%)
			–	315,533–314,805		
R9	692	99.86	+	80,337–81,028	783 (99.87%)	1 (0.13%)
			–	309,412–308,722		
R10	72	97.22	+	302,169–302,240	1362 (99.93%)	1 (0.07%)
			+	313,560–313,631

Recombination involving large repeats generally result in equimolar or nearly equimolar recombined molecules in the genome [58], as exemplified in *Silene latifolia*, *Silene vulgaris* [7], *Mimulus guttatus* [33], and Ginkgo [31]. In our study, two large repeats (out of 2224 repeats) with a length of 3293 bp and 1538 bp show evidence of recombination but with low recombination frequency at only 0.24% and 8.18%. Such low recombination frequency has also been seen in other plant mitogenomes, for example, in *Silene conica* and *Silene noctiflora* [7], tens of large repeats induced recombinations at a frequency around 5%; in *Ginkgo biloba*, a large repeat of 1.5 Kb recombined at a frequency of 9%; in fern *Ophioglossum californicum*, two large repeats with 4-Kb and 1-Kb induced recombinations at frequency of 24.5% and 0.1%, respectively [9]. In addition to these large repeats, all seven medium sized repeats of the *Nymphaea* mitogenome recombined even more rarely with recombination frequencies ranging from 0.11 to 0.34%, which is similar to the observation in the gymnosperms *Ginkgo biloba* and *Welwitschia mirabilis* [31], ferns [9], the flowering plants *Cucumis sativus* [13] and *Vigna angularis* [26], whereas significantly lower than those observed from *Viscum scurruloideum* [10], *Silene latifolia* and *Silene vulgaris*. For example, in *Viscum*, three medium-sized repeats result in recombination equilibrium, and another two recombined actively at frequencies of 11.2% and 38.7%.

The low recombination level of the *Nymphaea* mitogenome is further evidenced by a small number of recombined reads detected, i.e., only 74 reads were found to support alternative configurations resulted from repeat-mediated recombinations (Additional file 7: FASTA.fa), accounting for only 0.13% of the total reads (74 out of 56,849), which is apparently lower than those found in other plants, such as 10% in *Mimulus guttatus* [33], 6.6% in *Ophioglossum*, and 2.2% in *Psilotum*. The low level of recombination rate found in *Nymphaea* suggests the predominant existence of master configuration in vivo in this plant, with a low level of substoichiometric recombinant forms. The latter has been proved to exert profound effect on plant growth, such as cytoplasmic male sterility (CMS) [59–61] and abnormal growth phenotypes [62, 63].

Understanding the paradoxical coexistence of the low recombination and abundant repeats in mitogenomes, such as *Nymphaea* and *Ophioglossum,* must take into account the nucleus’ control over the accuracy of the repair of mitochondrial chromosomes by a series of nuclear-encoded and mitochondrial targeted factors [64, 65]. Disruption of these genes could initiate and promote mitochondrial intragenomic recombination [58], as have been documented in *Physcomitrella* [66] and *Arabidopsis* [24]. Such nuclear genes may be under different levels of selection pressure, resulting in distinctive stability of mitogenomes in specific plant groups. For example, in each of the major bryophyte lineage, mitochondrial genomes kept a high degree of structural conservation over long period of evolution [2], which is in contrast to the observations in *Silene vulgaris* [25] and *Beta vulgaris* [67] with remarkable intraspecific mitogenome rearrangements.

### Plastid DNA insertions

The *Nymphaea* mitogenome possesses 23 fragments of plastid derived sequences ranging from 38 bp to 1878 bp (Table 3) with a total length adding up to 13 Kb. The plastid derived sequences comprise 2% of the mitogenome, which is a typical percentage in angiosperms with the absolute amount of plastid inserts ranging from 4.4 Kb in *Arabidopsis* [68] to 138 Kb in *Amborella* [48]. Most (19 out of 23) of these plastid inserts, including those carrying tRNAs, having homologs in other plant mitogenomes, provides a good opportunity to revisit the origin of functional intracellular gene transfers, which remained ambiguous from seed plants [69] or vascular plants [1, 70]. Here we show evidence of the emergence of functional plastid insertions in ferns as exemplified by the presence of plastid derived functional tRNA gene *trnN*(*GTT*)-pt in fern *Ophioglossum*. Specifically, the 97-bp *Nymphaea* plastid insert carrying *trnN*(*GUU*)-pt have a 73-bp homolog in fern *Ophioglossum* (coverage 90%, identity 90%), in addition to a number of seed plants, suggesting the putative emergence of *trnN*(*GUU*)-pt in the ancestor of vascular plants, which is also evidenced by its extremely short flanking sequences measuring only a few bases due to long periods of purifying selections, given its relatively high sequence identity (92%) with their plastid counterparts. The plastid inserts in the *Nymphaea* mitogenome generally yielded similarities ranging from 74 to 97% (median 84%) while using *Nymphaea colorata* plastid genome sequence as a reference, indicating that most of the inserted sequences have been streamlined by the mitogenome and have accumulated considerable mutations. Particularly, in the *Nymphaea* mitogenome, the largest plastid insert of 1878 bp harboring *trnL*(*CAA*)-pt show comparatively conserved features with an identity of 96% in its tRNA region, which, however, rapidly declined to 88% and 83% in its up-stream and down-stream flanking sequences. Another two plastid inserts carrying *trnF*(*GAA*)-pt and *trnW*(*CAA*)-pt–*trnP*(*TGG*)-pt, respectively, also show similar degradation patterns in the flanking regions of the functional tRNA genes, as has been observed in *Liriodendron* [6]. The presence of the two plastid derived tRNAs including *trnF*(*GAA*)-pt and *trnL*(*CAA*)-pt in *Nymphaea*, *Amborella*, *Liriodendron*, several monocots, and some eudicots could possibly suggest their origin from the ancestor of angiosperms, followed by independent losses and/or gains during the evolution of angiosperms.

**Table 3:** Plastid insertions in the mitochondrial genome of *Nymphaea colorata*

Length (bp)	Position	Plastid genes contained	Identity (%)
1878	282,896–284,773	*trnL*(*CAA*)–*ndhB* (partial)	80.41
1438	328,952–330,389	*psaB* (partial)	84.87
1428	275,870–277,297	*ycf2* (partial)	87.60
1426	327,426–328,851	*psaB* (partial)–*psaA* (partial)	89.12
1194	592,277–593,470	*ndhJ*–*ndhK* (partial)	80.83
1059	299,952–301,010	*petG*(partial)–*trnW*(*CCA*)–*trnP*(*UGG*)–*psaJ*	85.62
857	412,364–413,220	16S rRNA (partial)	74.04
510	538,489–538,998	*ycf4* (partial)	81.88
447	11,109–11,555	*ndhF* (partial)	81.68
404	10,606–11,009	*ndhA* (partial)	90.59
380	330,507–330,886	*psaB* (partial)	78.74
370	278,097–278,466	none	80.67
346	79,612–79,957	16S rRNA (partial)	97.11
331	593,561–593,891	*trnF*(*GAA*)	84.10
268	277,340–277,607	*ycf2* (partial)	81.48
258	111,292–111,549	none	83.02
104	449,246–449,349	*trnQ*(*UUG*)	95.19
103	436,536–436,638	*trnS*(*UGA*) (partial)	83.50
97	135,004–135,100	*trnN*(*GUU*)	91.75
97	395,827–395,923	23S rRNA (partial)	81.63
78	132,465–132,542	*trnM*(*CAU*)	92.31
55	277,989–278,043	*ycf2* (partial)	94.55
38	328,916–328,953	*psaB* (partial)	94.87

### Conserved gene clusters

Plant mitogenomes are highly fluid in genome structure due to the repeat mediated homologous recombinations, sequence duplications, genome expansion and shrinkage, and incorporation of foreign DNAs [71], whereas some gene clusters are conserved across large phylogenetic scale [6, 50, 72]. The relatively low recombination level observed in *Nymphaea* does not necessarily predict strictly conserved genome arrangement compared with the ‘fossilized’ angiosperm mitogenome of *Liriodendron* or the other early angiosperm *Amborella*, as we found 38 and 44 rearrangements between mitogenomes of *Nymphaea*-*Amborella* and *Nymphaea*-*Liriodendron*, respectively. Nevertheless, in comparison of gene order of *Nymphaea* with that of the 214 plant mitogenomes, we identified 11 conserved gene clusters in *Nymphaea*, of which, three (*rpl2*–*rps19*–*rps3*–*rpl16*, *rps13*–*rps11*, and *rrn18*–*rrn5*) could be dated back deeply to the origin of mitochondrion from its endosymbiont bacterial ancestor [72]. The cluster *trnfM*(*CAU*)–*rrn26* is widely distributed in streptophytes. Four clusters (*cox3*–*sdh4*, *nad3*–*rps12*, *rpl5*–*rps14*–*cob*, and *rps10*–*cox1*) emerged since gymnosperms. The cluster *trnP*(*UGG*)–*sdh3* shows a sporadic distribution pattern in bryophytes, *Ginkgo*, *Cycas* and many angiosperms, indicative of the secondary loss of the gene cluster in lycophytes and ferns. The angiosperm conserved cluster *trnP*(*UGG*)-pt–*trnW*(*CAA*)-pt does not show up in *Amborella*, suggesting its emergence in *Nymphaea* or even earlier in the ancestor of angiosperms then secondary loss of the cluster in *Amborella*. The gene cluster <*nad5.× 4.× 5* > <*trnE* (*TTC*)–*nad7* > is only shared by three angiosperm species, namely, *Liriodendron*, *Nelumbo* and *Nymphaea*, suggesting its emergence in the ancestor of angiosperms followed by fast degeneration as a consequence of extensive genome rearrangements. However, the sporadic distribution of the cluster could more likely indicate a coincidence of independent structural evolutions in the three lineages (Additional file 8: Table S3).

## Conclusions

We assembled the complete mitogenome of *Nymphaea* using the PacBio RSII sequencing technology. *Nymphaea* mitogenome is similar to that of the *Amborella* and *Liriodendron* in the gene and intron contents, but significantly different in its abundant repetitive sequences. Whereas the recombination activity in the *Nymphaea* mitogenome is relatively quiescent, which evidenced by only a small portion of the examined reads*.* The length of plastid insertions of *Nymphaea* falls into the range of that of the other angiosperms, and some plastid derived tRNAs, with their existence in *Nymphaea* mitogenome, arguing for their earlier emergences in angiosperms than previously postulated. Finally, despite extensive genome rearrangements, 11 conserved gene clusters are identified in *Nymphaea*, which can be traced back to various stage of mitogenome evolution. This study shed new light on the evolution of mitochondrial genomes in early flowering plants, allowing deeper insights into the repeat-mediated recombination patterns in plant mitogenomes.

## Methods

### Mitochondrial genome assembly and annotation

The mitochondrial genome of *Nymphaea colorata* was obtained from the genome project of *Nymphaea colorata* led by Liangsheng Zhang (unpublished data). The genome sequencing was performed on a PacBio RSII platform (Pacific Biosciences, Menlo Park, CA). The Raw PacBio reads were corrected to accuracy above 99% using the RS_PreAssembler, and then assembled into contigs using the program Canu (github.com/marbl/canu). Two mitochondrial contigs of 527,532 bp and 157,672 bp were identified using the NCBI Blast program with the *Liriodendron* mitochondrial genome as a reference. The two contigs overlapped with each other at both ends by 34,745 bp and 16,132 bp, and finally formed a circular molecular of 617,195 bp, with an average depth of 601×. RNA-seq data of *Nymphaea colorata* were also obtained from the genome project of *Nymphaea colorata* (unpublished data).

The annotation for the *Nymphaea* mitogenome was performed as previously described [3, 70]. Protein coding genes and rRNA genes were annotated by blastn searches of the non-redundant database at National Center for Biotechnology Information (NCBI). The exact gene and exon/intron boundaries were further confirmed in Geneious software (v.10.0.2, Biomatters, www.geneious.com) by aligning each gene to its orthologs from available annotated plant mitochondrial genomes at the NCBI website (www.ncbi.nlm.nih.gov/genome/organelle). The tRNA genes were detected using tRNAscan-SE 2.0 [73].

### Repeats and repeat-mediated homologous recombinations

Repeats identification of *Nymphaea* and other vascular plant mitogenomes were carried out using NCBI blastn searches by searching the *Nymphaea* mitogenome sequence against itself with an e-value cut-off of 1e^− 6^, and a word size of 7 following Guo et al. [9]. All the repeat sequences were subsequently extracted and clustered into difference families using the program cd-hit-est as implemented in cdhit suite v4.6.7 [57], with a word size of 5 and sequence similarity threshold of 0.8. We estimated the number of repeats from the number of unique begin-end coordinates of hits from blastn search according to Alverson et al. [13]. To detect the active repeat-mediated intragenomic recombinations within the PacBio reads, we built up an mt read database using corrected genome sequencing PacBio reads. We used the *Nymphaea* mitogenome sequence as the reference to blast the total *Nymphaea* PacBio reads database with an e-value cut-off of 1e^− 100^ for extraction of mt reads, the resultant mt reads was further searched against *Nymphaea* plastid sequence with the same parameters to remove putative plastid reads with overall alignment coverage > 85% of the read length. Finally, we got a mitochondrial read database of 75,863 reads with an average length of 7294 bp, and total length 553,358,387 bp.

Repeat-mediated homologous recombinations were evaluated for those repeat pairs ranging from 30 to 3293 bp with blast identity > 80% following Alverson et al. [13]. Specifically, for each repeat pair, we built four or six reference sequences, each with 200 bp up- and down-stream of the two template sequences (original sequences), and the two (for repeat pair with identity =100) or four (for repeat pair with identity < 100) recombined sequences (alternative configurations) constructed from the putative recombination products. Then, we searched the reference sequences against the *Nymphaea* mt reads database, and count the number of matching reads with a blast identity above 99.5%, and a hit coverage over 200 bp in both flanking regions of each repeat sequence. After that, the templates with evidence of recombination were extracted and elongated in both sides to 2000 bp and searched again to the *Nymphaea* mt reads database to remove the recombinants with undersized flanking regions. Finally, the best matched reads for all the recombinants were extracted and aligned with the *Nymphaea* mitogenome in Geneious v10.0.2 (https://www.geneious.com/) to authenticate the accuracy of the recombined reads.

### Identification of plastid derived sequences

To identify plastid derived mitochondrial sequences, the *Nymphaea* mitogenome was searched against the plastid genome of *Nymphaea colorata* (data unpublished), and all plant mitogenome database with an e-value cut-off of 1e^− 6^ and a word size of 7, simultaneously. The blastn output was then visualized in Geneious v10.0.2 (https://www.geneious.com/) and each of the identified plastid sequence insert was compared with its co-occurring mt homologs from all other plant mitogenomes to infer the putative origin of the intracellular transfer.

### Identification of conserved gene clusters

The gene orders of *Nymphaea*, *Amborella*, and *Liriodendron* were compared with each other using UniMoG [74] to identify rearrangements among three mitogenomes. The conserved gene clusters were identified if they appeared in any two of the three early angiosperms and simultaneously presented in at least one major plant group, e.g., lycophytes, ferns, gymnosperms, or angiosperms.

## Additional files

Additional file 1: **Table S1.** Repeat proportions of the mitochondrial genomes of 82 angiosperm species. (PDF 96 kb)

Additional file 2: **Figure S3.** The DNA and RNA coverage plots of the cox2 gene of the mitochondrial genome of *Nymphaea colorata*. (PDF 139 kb)

Additional file 3: **Figure S4.** The conserved domain alignment of the group II intron cox2i373 of *Triticum timopheevii* (AP013106) and *Nymphaea colorata* (KY889142). (PDF 217 kb)

Additional file 4: **Table S2.** Eleven cis-spliced introns of the *Nymphaea* mitogenome with repeated sequences inserted. (PDF 66 kb)

Additional file 5: **Figure S1.** All the repeat pairs (886,982) evaluated for recombination in our study. The large number of repeats is due to numerous repeats that are partially overlapping with each other in Nymphaea mitochondrial genome. (a) The curve graph shows repeat distribution pattern on sequence identity. (b) The curve graph shows repeat distribution pattern on sequence length. (PDF 171 kb)

Additional file 6: **Figure S2.** The PacBio read depth plot of the mitochondrial genome of *Nymphaea* colorata. (PDF 96 kb)

Additional file 7: FASTA.fa. Seventy-four recombined reads detected for homologous recombination involving ten repeat pairs in our study. (FA 807 kb)

Additional file 8: **Table S3.** Eleven conserved gene clusters in the *Nymphaea* mitochondrial genome. (PDF 140 kb)

## Abbreviations

CMS: Cytoplasmic male sterility
HGT: Horizontal gene transfer
Mitogenome: Mitochondrial genome
mtDNA: Mitochondrial DNA
ORFs: Open reading frames
rRNAs: Ribosomal RNAs
tRNAs: Transfer RNAs
