# Supplementary material for: The complete mitochondrial genome of the early flowering plant Nymphaea colorata is highly repetitive with low recombination
Source: BMC Genomics. 2018 Aug 14;19:614. doi: 10.1186/s12864-018-4991-4 (PMC6092842; doi:10.1186/s12864-018-4991-4)
Supplement: Supplementary file 1 — Table S1. Repeat proportions of the mitochondrial genomes of 82 angiosperm species. (PDF 96 kb) [file 12864_2018_4991_MOESM1_ESM.pdf]

Table S1. Repeat proportions of the mitochondrial genomes of 82 angiosperm species.

Species	Small repeat proportion (%)	Medium repeat proportion (%)	Large repeat proportion (%)	Total repeat proportion (%)
Nymphaea colorata	3.97	28.1	16.82	48.89
Silene conica	20.85	10.71	10.47	42.03
Cucurbita pepo	27	11.1	0	38.1
Daucus carota	1.44	1.42	33.57	36.43
Tripsacum dactyloides	0.91	1.08	34.26	36.25
Gossypiumbarbadense	0.67	1.19	32.98	34.84
Zostera marina	3.8	9.54	20.33	33.67
Gossypium arboreum	0.73	1.29	31.03	33.05
Cucumis sativus	17.2	7.8	4.35	29.35
Gossypium raimondii	0.79	1.82	25.49	28.1
Rhazya stricta	0.8	0.52	25.58	26.9
Gossypium hirsutum	0.73	1.98	23.57	26.28
Nelumbo nucifera	2.1	6.41	17.32	25.83
Gossypium harknessii	0.74	2.07	22.94	25.75
Sorghum bicolor	1.07	1.09	21.69	23.85
Amborella trichopoda	9.1	13.8	0.14	23.04
Beta macrocarpa	1.63	3.44	17.28	22.35
Beta vulgaris	2	3.18	15.93	20.74
Zea mays	1.36	2	16.91	20.27
Hyoscyamus niger	1.62	4.63	13.81	20.06
Gossypium davidsonii	0.88	2.04	15.59	18.51

Gossypium trilobum	0.86	2.05	15.59	18.5
Glycine max	0.55	2.83	14.72	18.1
Nicotiana sylvestris	1.41	1.64	14.94	17.99
Nicotiana tabacum	1.41	1.63	14.95	17.99
Erythranthe guttata	0.92	0.98	15.65	17.55
Silene vulgaris	1.5	2.26	13.32	17.08
Capsicum annuum	1.79	1.73	13.55	17.07
Aegilops speltoides	0.99	1.54	14.51	17.04
Zea perennis	1.46	1.47	13.99	16.92
Triticum aestivum	1.25	1.75	13.83	16.83
Cocos nucifera	0.81	2.57	12.67	16.05
Triticum timopheevii	1.09	2.8	11.53	15.42
Lotus japonicus	0.87	1.9	12.3	15.07
Corchorus capsularis	5.81	3.25	5.76	14.82
Saccharum officinarum	0.74	0.54	13.01	14.29
Geranium maderense	3.65	10.11	0.45	14.21
Millettia pinnata	1.47	0.41	11.94	13.82
Liriodendron tulipifera	1.91	2.35	8.78	13.04
Raphanus sativus	2.15	3.12	7.53	12.8
Arabidopsis thaliana	1.39	5.21	5.88	12.48
Helianthus annuus	1.1	2.47	8.6	12.17
Sinapis arvensis	2.18	3.42	6.42	12.02
Spinacia oleracea	3.69	1.8	5.03	10.52
Stratiotes aloides	9.19	1.08	0	10.27
Zea luxurians	1.73	1.6	6.88	10.21
Brassica nigra	2.42	1.87	5.66	9.95
Brassica carinata	2.29	1.87	5.66	9.82
Utricularia reniformis	2.01	0.65	6.61	9.27
Butomus umbellatus	0.89	1.92	6.2	9.01
Ipomoea nil	1.98	6.53	0	8.51
Hibiscus cannabinus	1.14	2.98	4.38	8.5

Carica papaya	1.53	1.97	4.66	8.16
Lagerstroemia indica	5.99	2.09	0	8.08
Salvia miltiorrhiza	1.54	2.67	3.56	7.77
Brassica napus	2.23	2.76	2.19	7.18
Malus domestica	1.36	2.91	2.89	7.16
Citrullus lanatus	1.58	1.73	3.84	7.15
Brassica juncea	2.34	2.24	2.21	6.79
Brassica rapa	2.34	2.24	2.21	6.79
Salix suchowensis	1.13	0.75	4.84	6.72
Allium cepa	2.54	1.51	2.2	6.25
Silene latifolia	0.67	0.42	4.93	6.02
Diplostephium hartwegii	1.35	3.47	1.04	5.86
Castilleja paramensis	1.11	1.17	3.55	5.83
Vaccinium macrocarpon	1.86	2.43	1.21	5.5
Hesperelaea palmeri	1.09	1.38	2.42	4.89
Vitis vinifera	1.38	2.35	0.5	4.23
Ziziphus jujuba	1.31	2.25	0	3.56
Viscum album	2.11	1.44	0	3.55
Vigna angularis	0.87	2.46	0	3.33
Cannabis sativa	1.6	1.62	0	3.22
Asclepias syriaca	0.74	2.07	0.36	3.17
Spirodela polyrhiza	1.08	2.05	0	3.13
Dorcoceras hygrometricum	0.8	1.63	0.58	3.01
Phoenix dactylifera	0.89	1.79	0.33	3.01
Batis maritima	1.46	1.4	0	2.86
Populus davidiana	1.23	1.33	0	2.56
Medicago truncatula	1.51	0.52	0	2.03
Salix purpurea	1.07	0.91	0	1.98
Vigna radiata	0.92	1.06	0	1.98
Ajuga reptans	0.91	0.37	0	1.28
