# Supplementary material for: The complete mitochondrial genome of the early flowering plant Nymphaea colorata is highly repetitive with low recombination
Source: BMC Genomics. 2018 Aug 14;19:614. doi: 10.1186/s12864-018-4991-4 (PMC6092842; doi:10.1186/s12864-018-4991-4)

Figure S3. The DNA and RNA coverage plots of the *cox2* gene of the mitochondrial genome of *Nymphaea colorata* (KY889142).

The PacBio DNA read coverage plot

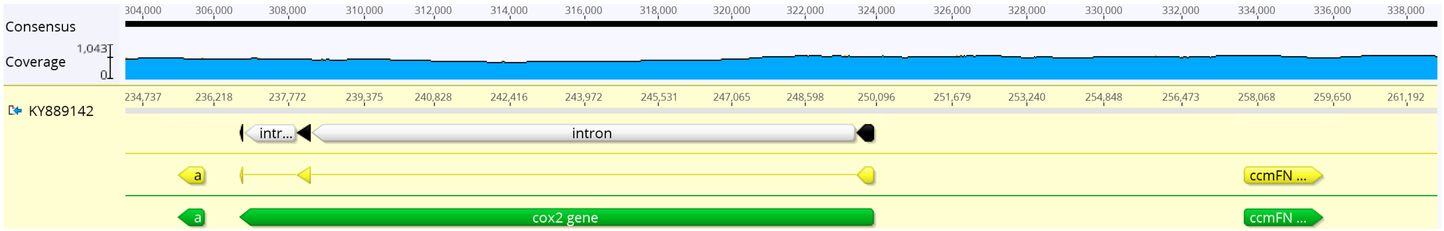

The RNA-seq read coverage plot

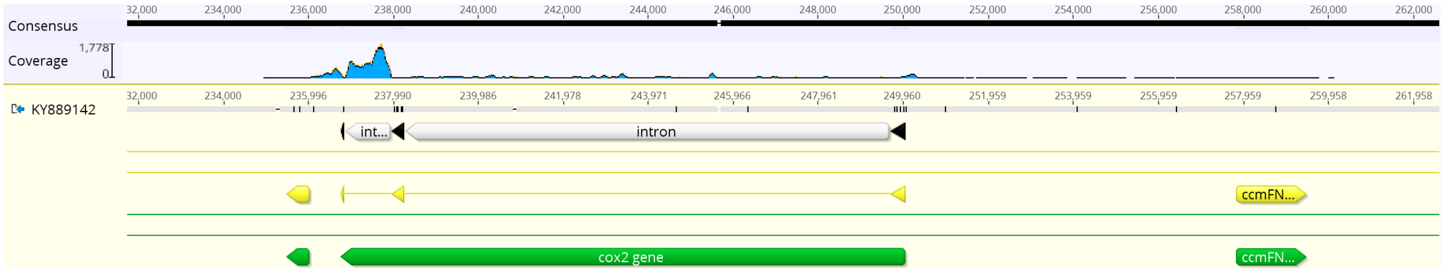

Supplement: Supplementary file 2 — Figure S3. The DNA and RNA coverage plots of the cox2 gene of the mitochondrial genome of Nymphaea colorata. (PDF 139 kb) [file 12864_2018_4991_MOESM2_ESM.pdf]
