# Supplementary material for: The complete mitochondrial genome of the early flowering plant Nymphaea colorata is highly repetitive with low recombination
Source: BMC Genomics. 2018 Aug 14;19:614. doi: 10.1186/s12864-018-4991-4 (PMC6092842; doi:10.1186/s12864-018-4991-4)

Figure S4. The conserved domain alignment of the group II intron cox2i373 of *Triticum timopheevii* (AP013106) and *Nymphaea colorata* (KY889142).

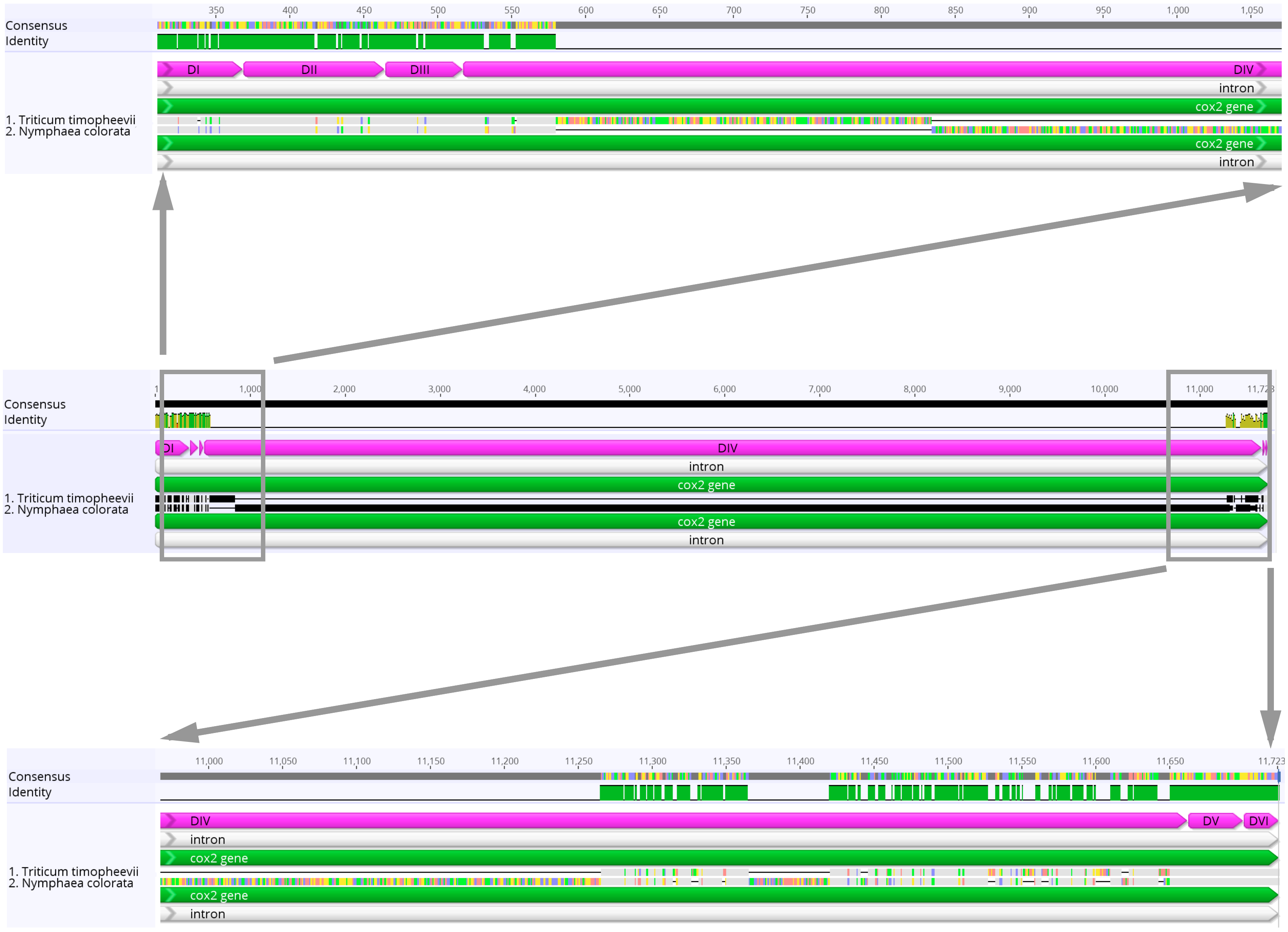

Supplement: Supplementary file 3 — Figure S4. The conserved domain alignment of the group II intron cox2i373 of Triticum timopheevii (AP013106) and Nymphaea colorata (KY889142). (PDF 217 kb) [file 12864_2018_4991_MOESM3_ESM.pdf]
