# Supplementary material for: The complete mitochondrial genome of the early flowering plant Nymphaea colorata is highly repetitive with low recombination
Source: BMC Genomics. 2018 Aug 14;19:614. doi: 10.1186/s12864-018-4991-4 (PMC6092842; doi:10.1186/s12864-018-4991-4)
Supplement: Supplementary file 4 — Table S2. Eleven cis-spliced introns of the Nymphaea mitogenome with repeated sequences inserted. (PDF 66 kb) [file 12864_2018_4991_MOESM4_ESM.pdf]

Table S2. Eleven cis-spliced introns of *Nymphaea* mitogenome with repeated sequences inserted.

Introns	Intron range	Intron length (bp)	Inserted repeat length (bp)	Repeat ratio (%)
cox2i373	238,262–249,672	11,411	9,072	79.50%
rpl2i846	467,959–474,891	6,933	5,486	79.13%
nad4i976	318,665–324,694	6,030	2,627	43.57%
nad2i1282	336,546–341,193	4,644	2,379	51.23%
nad2i156	583,586–587,604	4,019	2,806	69.82%
nad7i917	75,408–78,810	3,403	1,533	45.05%
rps3i74	464,431–466,848	2,418	389	16.09%
nad1i477	428,566–430,304	1,739	209	12.02%
rps10i235	102,192–103,618	1,427	204	14.30%
nad5i1872	58,554–59,720	1,167	175	15.00%
cox2i691	236,845–237,951	1,107	121	10.93%
