# Supplementary material for: The complete mitochondrial genome of the early flowering plant Nymphaea colorata is highly repetitive with low recombination
Source: BMC Genomics. 2018 Aug 14;19:614. doi: 10.1186/s12864-018-4991-4 (PMC6092842; doi:10.1186/s12864-018-4991-4)
Supplement: Supplementary file 5 — Figure S1. All the repeat pairs (886,982) evaluated for recombination in our study. The large number of repeats is due to numerous repeats that are partially overlapping with each other in Nymphaea mitochondrial genome. (a) The curve graph shows repeat distribution pattern on sequence identity. (b) The curve graph shows repeat distribution pattern on sequence length. (PDF 171 kb) [file 12864_2018_4991_MOESM5_ESM.pdf]

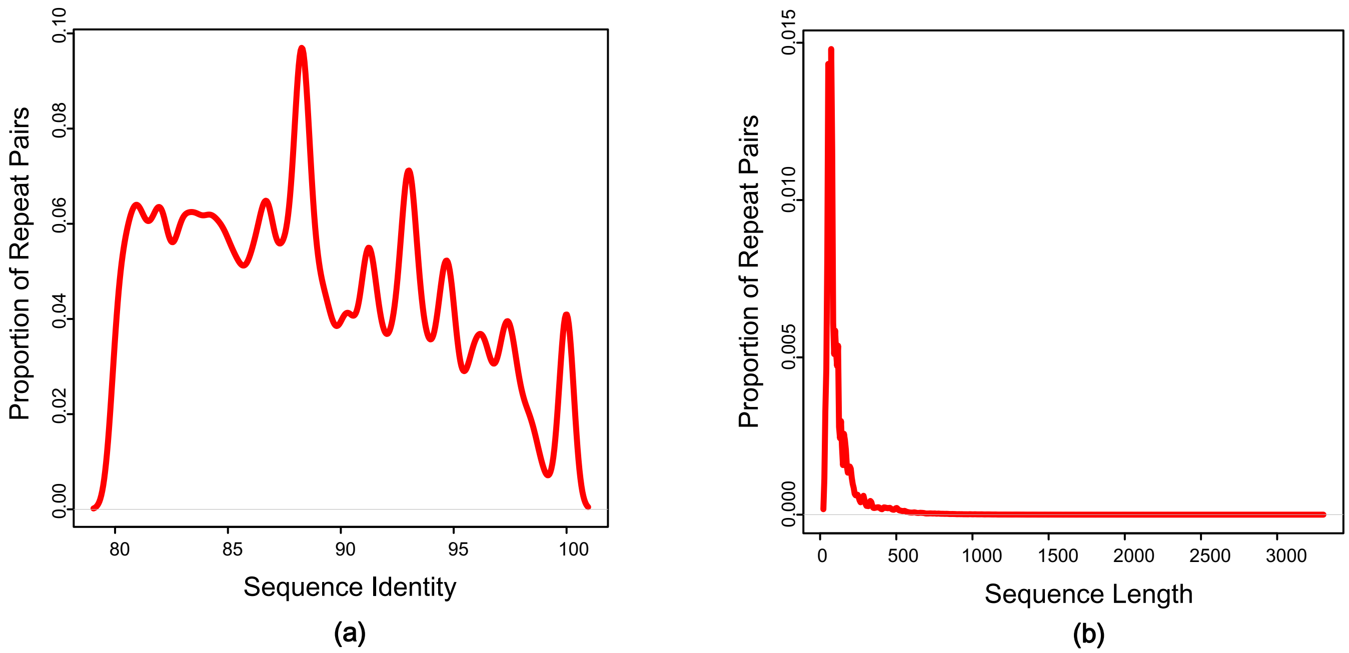

Figure S1. All the repeat pairs (886,982) evaluated for recombination in our study. The large number of repeats is due to numerous repeats that are partially overlapping with each other in *Nymphaea* mitochondrial genome. (a). The curve graph shows repeat distribution pattern on sequence identity. (b). The curve graph shows repeat distribution pattern on sequence length.
