# Supplementary figures and images for: The complete mitochondrial genome of the early flowering plant Nymphaea colorata is highly repetitive with low recombination
Source: BMC Genomics. 2018 Aug 14;19:614. doi: 10.1186/s12864-018-4991-4 (PMC6092842; doi:10.1186/s12864-018-4991-4)

**Figure S2. The PacBio read depth plot of the mitogenome of *Nymphaea colorata* (KY889142).**

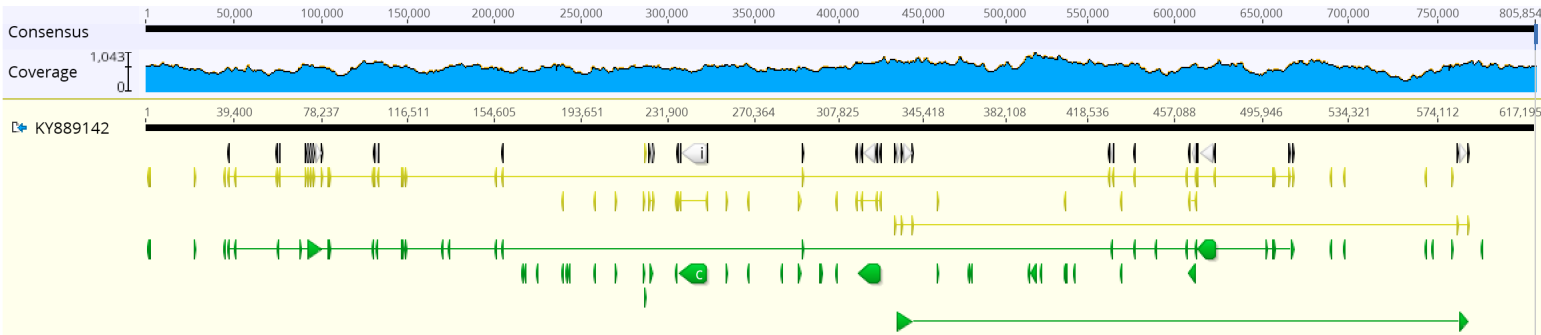

Supplement: Supplementary file 6 — Figure S2. The PacBio read depth plot of the mitochondrial genome of Nymphaea colorata. (PDF 96 kb) [file 12864_2018_4991_MOESM6_ESM.pdf]
