# Supplementary material for: The complete mitochondrial genome of the early flowering plant Nymphaea colorata is highly repetitive with low recombination
Source: BMC Genomics. 2018 Aug 14;19:614. doi: 10.1186/s12864-018-4991-4 (PMC6092842; doi:10.1186/s12864-018-4991-4)
Supplement: Supplementary file 8 — Table S3. Eleven conserved gene clusters in the Nymphaea mitochondrial genome. (PDF 140 kb) [file 12864_2018_4991_MOESM8_ESM.pdf]

Table S3. Eleven conserved gene clusters in *Nymphaea* mitochondrial genome.

No.	Conserved gene clusters	Distribution[a]
1	rpl2–rps19–rps3–rpl16	Bacteria
2	rps13–rps11	Bacteria
3	rrn18–rrn5	Bacteria
4	trnfM(CAT)–rrn26	Streptophytes
5	cox3–sdh4	Seed plants
6	nad3–rps12	Seed plants
7	rpl5–rps14–cob	Seed plants
8	rps10–cox1	Seed plants
9	trnP(TGG)–sdh3	Viridiplantae to basal angiosperms
10	trnP(TGG)-cp–trnW(CAA)-cp	Angiosperms but not in *Amborella*
11	<nad5.x4.x5><trnE(TTC)–nad7>	*Nymphaea*, *Liriodendron*, and *Nelumbo*

[a] exceptions are tolerated in each of the distribution range listed below.
